# The New *International Journal of Environmental Research and Public Health* (*IJERPH*) Policy Concerning Tobacco Company Funding

**DOI:** 10.3390/ijerph15122831

**Published:** 2018-12-12

**Authors:** The IJERPH Editorial Office

*IJERPH* is proud to announce that it is adopting a new policy concerning the publication of research funded by tobacco companies. As of 1 November 2018, the journal will no longer review or accept papers funded by tobacco companies or their subsidiaries as well as by groups supported by company funding. Specifically:

Tobacco Industry funding: *IJERPH* will not consider for publication papers reporting work funded, in whole or in part, by a tobacco company or tobacco industry organization or affiliate. Nor will the journal consider papers by authors who accept tobacco industry or affiliate funding, including funding for research costs and for all or part of any author’s salary, or other forms of personal remuneration.

E-cigarette Industry funding: With regards to research performed with funding, either directly or indirectly, from the e-cigarette industry (such as a grant from a company involved in the production or distribution of e-cigarette products), we request the full disclosure of such funding in the acknowledgments section of the manuscript. By “e-cigarette industry” we refer to companies or organizations engaged in designing, manufacturing, distributing, marketing, or promoting electronic nicotine-delivery systems.

Pharmaceutical industry funding: The same full disclosure approach as with e-cigarette funding is requested by research funded in part or in full by the pharmaceutical industry.

This policy is based on a combination of the policies and language adopted by the leading tobacco-focused journals *Tobacco Control*, *Tobacco-Induced Diseases*, and *Tobacco Prevention and Cessation*. Additionally, several other journals have adopted similar policies, including *PLoS Medicine*, *PLoS Biology*, *PLoS ONE*, the *British Medical Journal*, and journals published by the American Thoracic Society (https://www.atsjournals.org/). However, a few journals publishing tobacco control research (e.g., *Nicotine and Tobacco Research*) as well as public health journals (e.g., *American Journal of Public Health*) do not rule out reviews and publications of tobacco company-funded research.

This policy highlights several ethical issues in scientific publication cogently discussed by the *PloS Medicine* editors in 2010 when they adopted a similar policy [1] and the Editor of *Tobacco Control* which took similar steps in 2012 [2]. These issues include balancing the value of open discourse and transparency in scientific publication with the well-documented history of the tobacco industry’s efforts to prevent regulation by subverting scientific research to its own ends. Because of the enormous and ongoing public health importance of tobacco control worldwide, the journal remains highly interested in the review and publication of articles concerning diverse aspects of tobacco research, including environmental, policy, and regulatory approaches to understanding tobacco use, exposure to tobacco, and tobacco influences on health.

However, in our view, the history of tobacco companies’ misuses of science, exemplified by a 2006 U.S. federal court ruling that found that major cigarette manufacturers fraudulently covered up the health risks associated with smoking, creates a unique challenge to the value of openness [3]. This ruling resulted in the publication of a series of corrective statements, highlighting areas in which the company had deliberately attempted to deceive the public concerning the addictive nature of smoking and nicotine and the adverse health effects of smoking and secondhand smoke [4].

In summary, we believe this policy is appropriate for a journal dedicated to environmental and public health and formally announce that it is effective as of 1 November 2018.
